# Microfocused Ultrasound With Visualization in Skin Quality: A Narrative Review

**DOI:** 10.1111/jocd.70364

**Published:** 2025-08-23

**Authors:** Tatjana Pavicic, Jeremy B. Green, Je‐Young Park, Gabriela Casabona, Vasanop Vachiramon, Julieta Spada, Jennifer Levine, Mariana Muniz, John Akers, Alec McCarthy, Matthew J. Jackson

**Affiliations:** ^1^ Private Practice for Dermatology and Aesthetics Dr. Tatjana Pavicic Munich Germany; ^2^ Skin Research Institute and Skin Associates of South Florida Coral Gables Florida USA; ^3^ Apkoo‐Jung Oracle Dermatology Center Seoul Korea; ^4^ Ocean Clinic Marbella Málaga Spain; ^5^ Division of Dermatology, Faculty of Medicine Ramathibodi Hospital Mahidol University Bangkok Thailand; ^6^ Julieta Spada Dermatology and Aesthetics Buenos Aires Argentina; ^7^ Lenox Hill Hospital and Manhattan Eye, Ear, and Throat Hospital New York New York USA; ^8^ Dermatology Private Office São Paulo Brazil; ^9^ Global Medical Affairs, Merz Aesthetics Raleigh North Carolina USA

**Keywords:** emergent perceptual categories, energy‐based devices, MFU‐V, microfocused ultrasound with visualization, skin quality, Ultherapy

## Abstract

**Background:**

Skin quality is a multidimensional concept encompassing four emergent perceptual categories (EPCs): firmness, surface evenness, tone evenness, and glow. Microfocused Ultrasound with Visualization (MFU‐V; Ulthera System) is a non‐invasive device FDA‐cleared for lifting and tightening of the skin in specific areas. Through the generation of thermal coagulation points at defined depths, MFU‐V initiates neocollagenesis and elastin remodeling, which may support improvements in features related to overall skin quality.

**Aims:**

To evaluate clinical and preclinical evidence for the impact of MFU‐V across the four EPCs of skin quality, including firmness, surface evenness, tone evenness, and glow.

**Patients/Methods:**

A structured narrative review was conducted across PubMed, Google Scholar, and Cochrane databases. Of 703 unique records screened, 67 studies met inclusion criteria. Eligible studies evaluated MFU‐V, alone or in combination, reporting outcomes mapped to one or more EPCs of skin quality.

**Results:**

MFU‐V demonstrated consistent improvements in firmness (*n* = 52 studies), including elasticity and tautness, with sustained effects beyond 6 months. Improvements in surface evenness (*n* = 35) included wrinkle reduction, pore refinement, and scar remodeling. Effects on tone evenness (*n* = 4) were observed in melasma and erythema models, with good tolerance in Fitzpatrick skin types III–VI. Glow (*n* = 4) was indirectly supported by improved texture and structure. Combination protocols involving MFU‐V and fillers or neuromodulators reported positive outcomes. Adverse events were rare and transient.

**Conclusions:**

MFU‐V provides a safe and versatile platform for enhancing multiple aspects of skin quality. Its effects are well supported in firmness and texture, with emerging evidence for tone and glow. The integration of EPC‐based outcomes may inform individualized treatment planning and future research.

## 1 Introduction

Skin Quality is a multidimensional concept encompassing four emergent perceptual categories (EPCs): skin firmness, surface evenness, tone evenness, and glow. These perceptual domains reflect the composite visual and tactile cues that contribute to the appearance of healthy, undamaged skin [1]. Firmness relates to elasticity, structure, and resilience; surface evenness reflects smoothness, pore visibility, and the presence of lines or crepiness; tone evenness refers to uniformity in pigmentation and vascular appearance; and glow describes the overall luminosity and vitality of the skin, often shaped by the balance of the other three EPCs. Improvements in one domain usually influence others, underscoring the interconnected nature of skin quality [1]. Improvements in one domain often influence others, underscoring the interconnected nature of skin quality.

**FIGURE 1:**
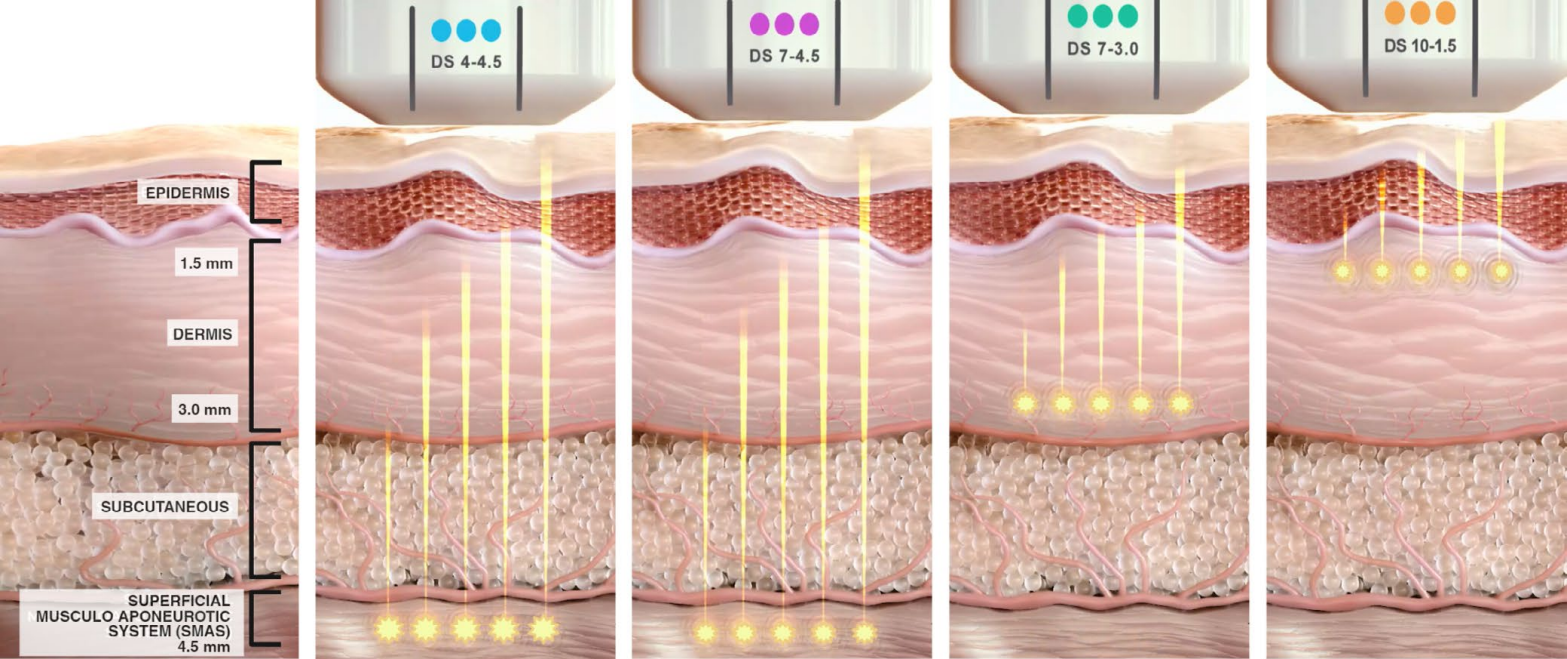
MFU‐V transducers targeting various skin layers (SMAS, reticular/papillary dermis) at specific depths (4.5, 3.0, 1.5 mm), demonstrating precise depth‐controlled thermal coagulation. DS, DeepSee. Figure reproduced from: Vachiramon et al. *J Cosmet Dermatol*. 2025; 24 (2):e16658. Licensed under CC BY 4.0.

Microfocused ultrasound with visualization (MFU‐V, Ulthera System, Ulthera Inc., Raleigh, NC, USA, a company of the Merz Aesthetics group) is a non‐invasive energy‐based device designed to deliver focused ultrasound energy at precise depths in the dermis and subdermal tissue. The energy generates discrete thermal coagulation points (TCPs) [2, 3, 4]. These localized thermal zones initiate tissue remodeling, including collagen contraction and reorganization, leading to lifting and firming effects without disrupting the skin surface [2, 5, 6, 7, 8]. MFU‐V distinguishes itself from other modalities by targeting collagen‐rich structures, such as the deep dermis and superficial musculoaponeurotic system (SMAS), at depths of 1.5, 3.0, and 4.5 mm, achieving therapeutic temperatures between 60°C and 70°C, which are optimal for neocollagenesis (Figure 1) [9, 10, 11, 12, 13].

A key differentiator of MFU‐V is its real‐time ultrasound imaging, which allows clinicians to visualize skin layers during treatment. This enables precise energy delivery to appropriate tissue depths while avoiding sensitive structures like bone and vessels, enhancing both safety and efficacy [13, 14], supporting tailored treatment based on patient anatomy, increasing consistency and confidence in clinical outcomes.

**FIGURE 2:**
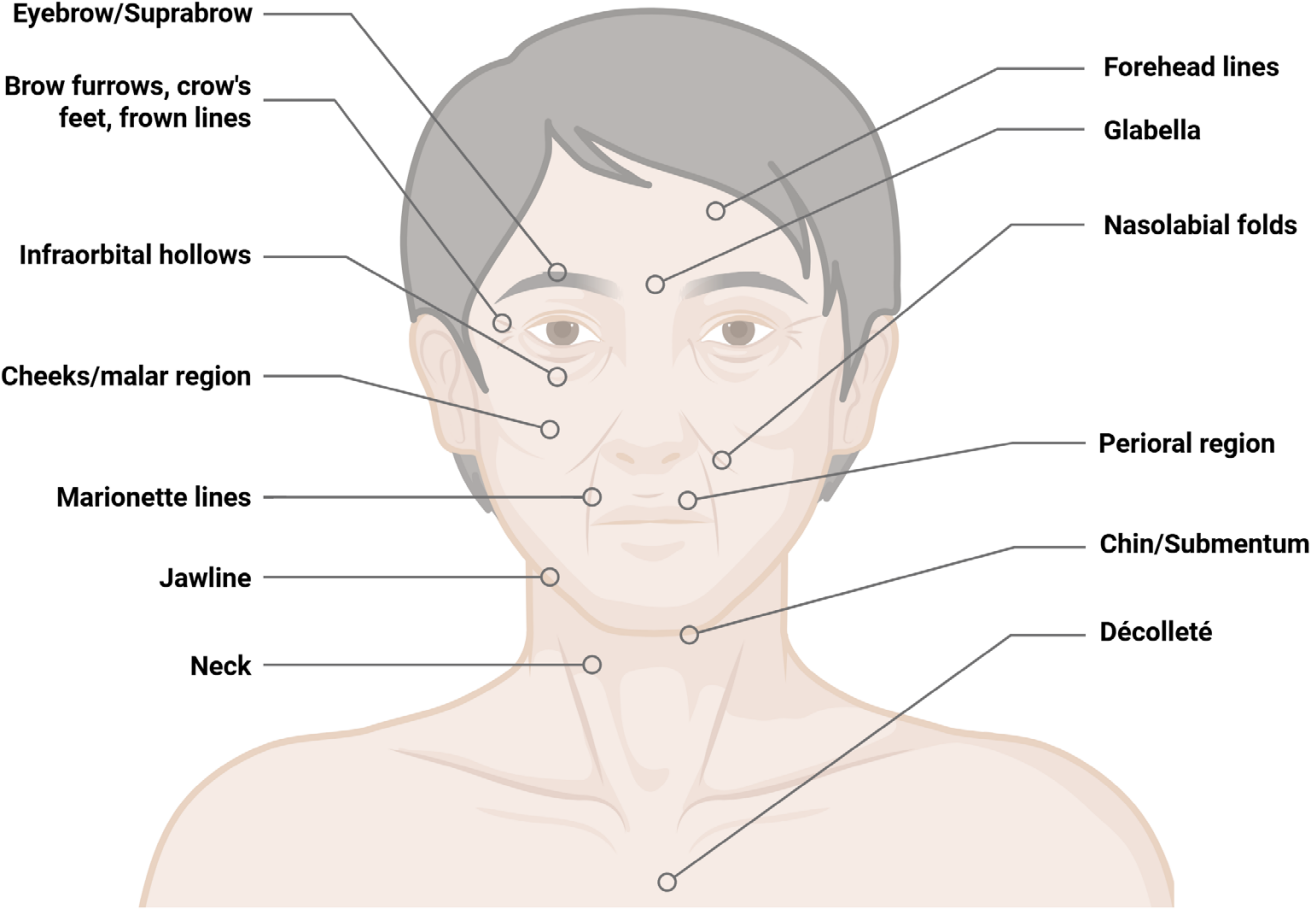
Common aesthetic concern areas of the face and chest.

MFU‐V was first FDA‐cleared in 2009 for non‐invasive brow lifting and has since gained additional clearances for neck and submental lifting, as well as for improving lines and wrinkles on the décolletage (Figure 2) [9]. It remains the only microfocused ultrasound device with FDA clearance for visualization and treatment. Across clinical trials and expert consensus, MFU‐V has demonstrated a strong safety profile, including in patients with Fitzpatrick skin types III–VI, due to its ability to bypass the melanin‐rich epidermis and minimize the risk of post‐inflammatory hyperpigmentation [1, 15, 16, 17, 18, 19]. Reported adverse events (AEs) are typically mild and transient, such as erythema or tenderness [10, 11, 15, 16, 17, 20, 21, 22, 23, 24, 25, 26].

This narrative review evaluates the evidence for MFU‐V's impact across the four EPCs of skin quality: firmness, surface evenness, tone evenness, and glow. Findings from pre‐clinical studies, clinical trials, and patient‐reported outcomes are integrated to explore its role in supporting structural and aesthetic skin improvements.

## 2 Methods

This narrative review followed a structured search and screening process to identify clinical and preclinical studies evaluating the effects of MFU‐V on skin quality [27]. The goal was to capture all relevant evidence addressing one or more of the four EPCs of skin quality: firmness, surface evenness, tone evenness, and glow (Table 1) [1].

**TABLE 1:** MFU‐V evidence across skin quality EPCs.

EPC	Key parameter	Effect of MFU‐V	Strength of evidence (no. studies)
Firmness (*n* = 52)	Tautness/Tightness	Consistent improvement across face, neck, and body; enhanced with combinations	High (*n* = 43)
	Elasticity	Mixed results; improved with monotherapy and combinations in some studies	Moderate (*n* = 14)
	Hydration	Maintains epidermal hydration and barrier integrity without compromising function	Low (*n* = 1)
Surface evenness (*n* = 35)	Wrinkles/Lines	Reductions are seen especially in the periocular, neck, and décolletage areas	High (*n* = 23)
	Crepiness	Improvement in fine, crinkled skin texture in delicate areas (neck, periocular, décolletage); enhanced with combos	Moderate (*n* = 6)
	Pore Size	Significant reduction with MFU‐V alone and with HA	Moderate (*n* = 3)
	Scars	Atrophic acne and striae scars improved with MFU‐V, especially in combination with CaHA and microneedling	Moderate (*n* = 3)
Tone evenness (*n* = 4)	Pigmentation	Improvement in UV and melasma models, especially in skin types III–IV	Moderate (*n* = 3)
	Erythema	Reduction in rosacea	Low (*n* = 1)
	Coloration	Improvement in melasma with significant lightening	Low (*n* = 1)
Glow	n/a	Indirect benefits via improved tone, texture, and smoothness	Low (*n* = 4)

*Note:* This table summarizes the impact of Microfocused Ultrasound with Visualization (MFU‐V) on skin quality, categorized by Emergent Perceptual Categories (EPCs) and their key parameters. The strength of evidence is described as follows; High—Strong and consistent clinical evidence from multiple studies, often supported by objective measures; Moderate—Generally supportive findings, though some variability exists in study design or outcomes; Low–Moderate—Preliminary or limited data; effects are promising but not yet well established; Emerging—Subjective or perceptual outcomes (e.g., glow or radiance) not yet standardized or extensively studied. All categorized studies are provided in Table 3.

Abbreviations: CaHA, calcium hydroxylapatite; HA, hyaluronic acid; PIH: post‐inflammatory hyperpigmentation.

Electronic searches were performed across PubMed (*n* = 508), Google Scholar (*n* = 268), and the Cochrane Central Register of Controlled Trials (*n* = 44). After deduplication (*n* = 117), 703 records were screened. A tiered exclusion process was applied, beginning with automated filtering of metadata to remove publications that were review articles, editorials, letters, or commentaries (*n* = 143), non‐MFU‐V focused (*n* = 179), not skin quality–relevant (*n* = 146), or unrelated to aesthetic dermatology or underlying conditions (*n* = 95). This left 140 full‐text articles for manual review. These studies were screened based on full content, resulting in a final inclusion of 67 studies that addressed the impact of MFU‐V on one or more skin quality domains (Table 2).

**TABLE 2:** Summary of manuscript search and screening process.

Stage	Count
Records identified via PubMed	508
Records identified via Google Scholar	268
Records identified via Cochrane Central Register of Controlled Trials	44
**Total records identified**	**820**
Duplicates removed	117
**Records screened after deduplication**	**703**
Records excluded via automated metadata filtering	
Review articles, editorials, letters, or commentaries	143
Not MFU‐V focused	179
Not skin quality–relevant	146
Unrelated to aesthetic dermatology or underlying conditions	95
**Full‐text articles assessed for eligibility**	**140**
Full‐text articles excluded	73
**Studies included in qualitative synthesis (narrative review)**	**67**

The search strategy and Boolean operators used are outlined in the Supporting Information. Articles were eligible if they included MFU‐V interventions—alone or in combination—with clear outcome measures mapped to the EPCs. Preclinical, clinical, histological, and patient‐reported outcome data were all eligible for inclusion. A summary of each study grouped by EPC domain is shown in Table 3.

**TABLE 3:** Summary of studies evaluating MFU‐V across skin quality domains.

Study	Design	Population	Treated sites	Treatment	Outcomes
Firmness					
Elasticity					
Casabona and Michalany (2014) [4]	Clinical and histological evaluation	*N* = 1; Female = 100%; Ethnicity: n/s	Inner thigh and retroauricular area	Single session MFU‐V (4.5‐, 3.0‐, 1.5‐mm) combined with CPM‐HA and CaHA‐CMC fillers	Significant enhancement in number and quality of collagen and elastin fibers, leading to thickening and denser fibers and contributing to 3‐dimensional rejuvenation
Casabona et al. (2023) [28]	Case study	*N* = 1; Female = 100%; Ethnicity: n/s	Excess skin from the pre‐auricular region	Single session MFU‐V (1.5, 3.0, 4.5 mm), CaHA‐CMC, CPM‐HA 20G microneedling	Greatest improvements seen when MFU‐V administered immediately before fillers. Epidermal and dermal thickness increased 3‐fold in areas treated with MFU + CaHA + HA; visible septal thickening; collagen fibers showed parallel reorganization
Casabona (2020) [29]	Retrospective study	*N* = 48; Female = *n*/s; Ethnicity: n/a	Lower face and neck	Single session MFU‐V (1.5, 3.0, and 4.5 mm); 30 days after thread insertion	Merz Aging Face Scale scores decreased from 2.17 to 1.08 (lower face) and 2.15–1.15 (neck) at 90 days (*p* < 0.001); denser neocollagenesis with MFU‐V + thread versus MFU‐V alone; maintained at 1 year
Corduff and Lowe (2023) [21]	Pilot case study	*N* = 10; Female = 90%; Ethnicity: n/s	Full‐face and upper neck	Multi‐session MFU‐V using the Hi5 protocol	Improved skin and fibromuscular laxity, including improvements in skin elasticity, while enhancing patient comfort and affordability through staged sessions
Doh et al. (2018) [30]	Retrospective case series	*N* = 10; Female = 100%; Ethnicity: Korean	Lower neck	Single session MFU‐V, CPM‐HA filler, Incobotulinumtoxin‐A, and CaHA‐CMC (1 patient)	Significant reduction in skin laxity scores from 1.30 to 0.725 (*p* = 0.008); all 10 patients showed clinical improvement
Juhász et al. (2023) [31]	Pilot, open‐label, prospective, split‐body trial	*N* = 20; Female = 100%; Ethnicity: Caucasian, Black	Lower anterior thigh and knee	Dual‐depth or triple‐depth single session MFU‐V followed by dilute CaHA‐CMC (1:1) intradermally	Significant improvement in Merz Skin Laxity Scale for treated knees at 12 and 24 weeks (*p* = 0.001, *p* = 0.004)
Kerscher et al. (2019) [32]	Observational, single‐center, open‐label safety evaluation	*N* = 22; Female = 100%; Ethnicity: Caucasian	Lower face and submental region	Single session MFU‐V (4.5 and 3.0 mm depth)	Net skin elasticity significantly increased at 12 and 24 weeks compared to baseline (*p* < 0.05). Gross skin elasticity significantly increased at weeks 12 and 24
Kerscher et al. (2018) [33]	Rater‐blinded observational study	*N* = 22, 9 combination; Female = 100%; Ethnicity: Caucasian	Lower face and submental region	Single session MFU‐V. Subjects with insufficient response received 1.5 mL CaHA‐CMC filler at 12 weeks	Skin firmness improved at 48 weeks (*p* < 0.05); GAIS: 50% very much improved, 50% much improved. Skin thickness increased at 24 weeks (*p* < 0.05), reducing laxity
Kwon et al. (2018) [34]	Clinical evaluation	*N* = 22; Female = 100%; Ethnicity: Korean	Face	Single session MFU‐V session followed by 20% glucose injection in temporal areas	Firmness: 82% improvement at week 1, 91% improvement at 12 weeks; histological increase in dermal collagen
Lee et al. (2015) [20]	Prospective randomized clinical trial	*N* = 22; Female = 86%; Ethnicity: Korean	Cheek	Single session MFU‐V using either a 1.5‐mm or a 3.0‐mm transducer	Significant increases in gross elasticity (R2) and a positive correlation between elasticity improvement and pore reduction
Park et al. (2020) [35]	Pilot study	*N* = 20; Female = 100%; Ethnicity: Korean	Upper face	Single session MFU‐V (3.0 and 1.5 mm), CPM‐HA fillers, Incobotulinumtoxin‐A	Average eyebrow height increased from 2.91 to 3.27 cm and maximum from 3.26 to 3.64 cm at 12 weeks (*p* < 0.05). VAS scores for eyebrow ptosis and periorbital area improved significantly by week 12 (*p* < 0.05). GAIS scores improved to 3.7 (physician) and 3.8 (patient) by week 12
Ramirez et al. (2021) [36]	Prospective, open‐label, non‐randomized, single‐arm pilot study	*N* = 12; Female = 100%; Ethnicity: Caucasian, Asian	Brachial region	Single session MFU‐V (4.5 and 3.0 mm) + subdermal CaHA‐CMC (1:1 or 1:2 dilution)	Significant improvement in skin firmness (*p* < 0.05) and elasticity *p* < 0.05); 88% showed aesthetic improvement at 12 weeks
Sasaki et al. (2017) [14]	Randomized split‐face study	*N* = 20; Female = 90%; Ethnicity: Hispanic, Caucasian	Marionette folds	Single session Study 1: MFU‐V (4.5 and 3.0 mm) Study 2: MFU‐V (4.5 and 3.0 mm) versus 3 planes (adding 1.5 mm)	Study 2: Significant difference in elasticity after treatment of 2 versus 3 tissue planes at day 180 (*p* < 0.05) and D360 (*p* < 0.05)
Woodward et al. (2014) [37]	Retrospective analysis	*N* = 100; Female = *n*/s; Ethnicity: n/s	Face and neck	Single session MFU‐V (4.5‐ or 3.0‐mm), plus Fractional CO_2_ laser or eCO2 laser	Significant improvement in skin laxity was observed with the combination of MFU‐V + AFL treatment
Hydration					
Kerscher et al. (2019) [32]	Observational, single‐center, open‐label safety evaluation	*N* = 22; Female = 100%; Ethnicity: Caucasian	Lower face and submental region	Single session MFU‐V (4.5 and 3.0 mm depth)	Skin hydration values were ‘fairly stable’ and showed ‘no significant differences’ at short‐ and long‐term measurements compared to baseline
Tautness/Tightness					
Alhaddad et al. (2019) [38]	Randomized, prospective, single‐center, evaluator‐blinded, split‐face clinical trial	*N* = 20; Female = 100%; Ethnicity: n/s	Face and upper neck	Single session MFU‐V and MRF at Day 0	Both treatments reduced cheek laxity, nasolabial folds, and jowls. For upper neck laxity, both improved at Days 30 and 90; by Day 180, only the MFU‐V side showed significant change
Alster and Tanzi (2012) [39]	Prospective clinical evaluation	*N* = 18; Female = 100%; Ethnicity: n/s	Upper arms, medial thighs, and extensor knees	Single session MFU‐V; single‐plane (4.5‐mm) versus dual‐plane (4.5‐mm then 3.0‐mm)	All areas showed significant improvement (*p* < 0.001). Arms and knees showed more skin lifting and tightening than thighs. Dual‐plane areas had better scores and greater skin texture improvement
Araco (2020) [40]	Prospective study	*N* = 50; Female = 94%; Ethnicity: Caucasian, Asian	Face and neck	Single session MFU‐V (4.5, 3.0, and 1.5 mm), targeting the SMAS, subcutaneous tissue, and deep dermis	Scores showed significant improvement in face laxity and ptosis; doctors scored ptosis improvement over 80 points out of 100
Bartsch et al. (2020) [41]	Randomized interventional prospective study	*N* = 61; Female = 100%; Ethnicity: n/s	Gluteal and posterior thigh regions	Arm 1: TSGS (day 0) then MFU‐V (day 90). Arm 2: TSGS (day 0) then CaHA (day 90). Arm 3: TSGS (day 0) then MFU‐V + CaHA (day 90). Arm 4: MFU‐V + CaHA (day 0) then TSGS (day 90)	Modalities with all three improved skin laxity more (odds ratio 1.88; 95% CI, 0.66–5.37). The best combo was MFU/CaHA with TSGS after 3 months (Arm 4)
Baumann and Zelickson (2016) [42]	Nonrandomized, prospective study	*N* = 64 Female = 95% Ethnicity: Caucasian, Hispanic, African American	Submental, submandibular, lower neck, and platysmal areas	Single session MFU‐V Group A: Lower neck (3 mm depth), submental and submandibular areas (4.5 and 3 mm depths) Group B: Submental, submandibular, and lower neck (4.5 and 3 mm); single depth for platysmal muscle. Group C: Submental, submandibular, and lower neck (4.5 and 3 mm depths)	Improved skin laxity, neck folds, sagging, texture, and ptosis observed at 60, 90, and 180 days. Two focal depths showed slightly greater aesthetic improvement
Casabona and Michalany (2014) [4]	Clinical and histological evaluation	*N* = 1; Female = 100%; Ethnicity: n/s	Inner thigh and retroauricular area	Single session MFU‐V (4.5‐, 3.0‐, 1.5‐mm) combined with CPM‐HA and CaHA‐CMC fillers	Retroauricular sites showed denser, more organized collagen and elastin bundles following combination treatment compared to MFU‐V alone
Casabona and Pereira (2017) [43]	Retrospective study	*N* = 20; Female = 100%; Ethnicity: n/s	Buttocks and upper thighs	Single session MFU‐V using (4.5‐, 3.0‐mm) immediately followed by subdermal CaHA‐CMC	Significant improvements in skin laxity/flaccidity/sagging. Patient satisfaction included perceived skin changes and smoother skin
Casabona et al. (2023) [28]	Case study	*N* = 1; Female = 100%; Ethnicity: n/s	Excess skin from the pre‐auricular region	Single session MFU‐V (1.5, 3.0, 4.5 mm), CaHA‐CMC, CPM‐HA 20G microneedling	Increased SMAS thickness, better dermal adhesion via fibrous septae thickening; MFU‐V + fillers improved collagenous 3D matrix, enhancing structural stability
Casabona (2019) [44]	Retrospective, nonrandomized study	*N* = 20; Female = 100%; Ethnicity: n/s	Breasts (10%), Buttocks (15%), Thighs (35%), Abdomen (40%)	Single session MFU‐V (3.0 and 1.5 mm) in a cross‐hatch pattern; subjects previously treated with CaHA, 20% ascorbic acid, and microneedling	Collagen remodeling and improved dermal structure to support tautness mechanisms noted histologically
Chang et al. (2019) [45]	Retrospective single‐arm, single‐center survey study	*N* = 83; Female = 98%; Ethnicity: Caucasian, Asian, Hispanic, Other/multiracial	Full face, neck, chest, and body	Single session MFU‐V (4.5 and 3 mm), following Amplify Ultherapy protocol guidelines. Single‐depth MFU‐V (3 mm) occasionally used for thinner areas	37.3% reported mild, 27.7% moderate, and 14.5% significant improvement.
Chaves Bellote and Miot (2021) [46]	Case Series	*N* = 4; Female = 100%; Ethnicity: n/s	Buccinators region	Single session MFU‐(4.5‐mm and 3.0‐mm)	GAIS scores at 180 days: 3 participants ‘very improved’, 1 ‘improved’. All participants and the practitioner reported high satisfaction with facial slimming and improved contour
Cheng (2019) [47]	Case report	*N* = 1; Female = 100%; Ethnicity: n/s	Submental and submandibular areas	Single session MFU‐V (4.5‐ and 3‐mm) to submental/submandibular areas and lower cheek, with IncobotulinumtoxinA at lower middle mandible, platysma and to mouth corners	Improvement in mandibular angle and chin projection; enhanced jawline definition and straighter cervicomental contour maintained through 3 months
Corduff and Lowe (2023) [21]	Pilot case study	*N* = 10; Female = 90%; Ethnicity: n/s	Full‐face and upper neck	Multi‐session MFU‐V using the Hi5 protocol	Improvement in skin and fibromuscular ptosis and overall soft tissue laxity in all patients. Mean brow height increased by 1.7 mm, and mean submental lift was 78.7 mm^2^
Fabi et al. (2013) [18]	Single‐center, prospective cohort study	*N* = 21; Female = 100%; Ethnicity: FST I and II	Décolletage	Single session MFU‐V treatment (4.5‐ and 3.0‐mm depths)	Patient questionnaires reported improvement in tightening/lifting by 29% at day 90 and 24% at day 180
Fabi and Goldman (2014) [48]	Retrospective Evaluation	*N* = 45; Female = 100%; Ethnicity: Caucasian, Hispanic, Pacific Islander, Asian, Mixed	Face and upper neck	Single session MFU‐V (4.5‐ and 3.0‐mm depths)	Tightening lasted up to 180 days. GAIS scores showed 81.3% and 77.7% patient improvement at 90 and 180 days, respectively. Subject‐perceived improvement was 75% at 90 days and 77.8% at 180 days
Fabi et al. (2015) [49]	Prospective, open‐label, multicenter pilot clinical trial	*N* = 125; Female = 100%; Ethnicity: White, Hispanic/Latino, Other	Décolleté	Single session MFU‐V (4.5, 3.0, and 1.5 mm depths)	Subject‐reported reduction in sagging increased from 7% at 90 days to 49.5% at 180 days. Tighter/lifted skin was reported at 28.9% at 90 days and 4.4% at 180 days
Fusano et al. (2021) [50]	Comparative observational study	*N* = 54 women (27 vegan, 27 omnivore); Female = 100%; Ethnicity: FST II, III, and IV	Lower face and neck	Single session MFU‐V (4.5, 3, 1.5 mm depths)	At 3 months, both groups improved; vegans had less reduction in FLR for face (*p* = 0.04) and neck (*p* = 0.004). At 6 months, vegans showed worse outcomes on face (*p* = 0.001) and neck (*p* < 0.001) than omnivores
Gold et al. (2014) [51]	Prospective, open‐label, nonrandomized trial	*N* = 28; Female = 100%; Ethnicity: Caucasian, African American	Skin above the knees (bilateral)	Single session MFU‐V (4.5 mm then 3.0 mm depths)	Improved knee skin tightening seen in 86% of subjects at 90 and 180 days per PGAIS. SGAIS was 86% at 90 days, decreasing to 57% at 180 days. Satisfaction was 86% at 90 days, 68% at 180 days, matching perceived improvements
Goldberg and Hornfeldt (2014) [52]	Prospective, open‐label pilot study	*N* = 27; Female = 97%; Ethnicity: Caucasian, African American, Hispanic/Latino	Buttock	Single session MFU‐V (4.5 mm then 3.0 mm depths)	At 90 days, PGAIS showed 81.5% improvement, rising to 89.5% at 180 days. SGAIS was 74.1% at 90 days and 89.5% at 180 days. Subject satisfaction was 59.3% at 90 days and 68.4% at 180 days. Less sagging was reported by 59.3% at 90 days and 52.6% at 180 days
Harris and Sundaram (2015) [22]	Open‐label, nonrandomized clinical trial	*N* = 52; Female = 98%; Ethnicity: African American, Asian, Hispanic/Latino, Other	Facial and neck areas	Single session MFU‐V (4.5 and 3.0 mm depths)	The trial showed MFU‐V's safety and effectiveness in tightening and lifting skin for patients with darker skin types. Three temporary adverse events occurred, all resolving without lasting effects. While primarily focusing on safety, the study also showed improved skin laxity
Jones et al. (2017) [53]	Randomized, evaluator‐blinded, comparative trial	*N* = 20; Female = SMRF: 100%; MFU‐V: 90%; Ethnicity: n/s	Neck	Single treatment of SMRF or MFU‐V	Investigator: Both SMRF and MFU‐V significantly decreased neck laxity by Day 90, persisting to Day 180, with no significant difference between them. Subject‐assessed: Both improved laxity by Day 90, persisting to Day 180; MFU‐V showed earlier significant improvement
Jung et al. (2016) [54]	Evaluator‐blinded, split‐face study	*N* = 20 Female = 90% Ethnicity: Korean	Mid‐face region	MFU‐V versus HIFU: split face. Both, 4.5 mm depth	Qualitative assessments by clinicians and patients showed mild to moderate improvement in tautness/tightness. Quantitative assessment showed decreased values for both devices
Kerscher et al. (2019) [32]	Observational, single‐center, open‐label safety evaluation	*N* = 22; Female = 100%; Ethnicity: Caucasian	Lower face and submental region	Single session MFU‐V (4.5 and 3.0 mm depth).	Significant improvement in viscoelastic properties (net and gross elasticity) at 12 and 24 weeks
Lee et al. (2012) [55]	Prospective study	*N* = 10; Female = 100%; Ethnicity: FST III and IV	Face and neck	Single session MFU‐V (4.5‐mm then 3.0‐mm depths)	Clinicians: 80% of subjects showed skin tightening 90 days post‐treatment, with 25% significant, 50% moderate, and 25% mild. Self‐assessments: 90% subjective progress, including 20% significant, 50% moderate, and 20% mild
Lin (2020) [56]	Prospective study	*N* = 20; Female = 100%; Ethnicity: Asian, Caucasian	Lower abdomen	Single session MFU‐V (4.5‐, 3.0‐, and 1.5‐mm depth)	Significant improvements in investigator‐ and patient‐reported skin laxity scales (*p* < 0.001). Increased collagen and thicker fibrous septae. Patients felt less overweight (26%), embarrassed (23%), self‐conscious (16.5%), and bothered by abdominal appearance (23%). Patients were 30.5% happier with their appearance
Lowe (2021) [23]	Single‐center, Prospective case series	*N* = 9; Female = 100%; Ethnicity: n/s	Periorbital, perioral, accordion lines	Single session MFU‐V treatment (1.5 mm)	At 180 days, clinicians and subjects noted visible improvements in lines, especially around the periorbital and accordion areas, with high satisfaction
Lu et al. (2017) [57]	Single‐site, prospective, nonrandomized clinical trial	*N* = 25; Female = 92%; Ethnicity: Asian	Face, neck	Single session MFU‐V lines (4.5 mm for cheeks/neck; 3.0 mm for forehead/temple/cheeks/neck/infraorbital)	Significant submental lift at 90 days (*p* = 0.0217), decreasing at 180 days (*p* = 0.243). Mean brow height lifted 0.47 mm at 90 days (*p* = 0.0165), then down to 0.12 mm at 180 days (*p* = 0.6494). Physician and subject GAIS showed over 80% improvement at 90 and 180 days
Oni et al. (2014) [19]	Prospective, nonrandomized clinical trial with masked evaluation	*N* = 93; Female = 85%; Ethnicity: FST I‐VI	Lower face	Single session MFU‐V (4.5 mm then 3.0 mm depths)	Improved skin laxity in 58.1% of patients, with overall improvement in 63.6%. At 90 days, 65.6% perceived improvement in their lower face/neck. Quantitative assessment showed a 45.2 mm^2^ lift in 71.8%
Pak et al. (2014) [58]	Pivotal clinical trial	*N* = 7; Female = 100%; Ethnicity: Asian	Lower eyelids	Single session MFU‐V (1.5 and 3.0 mm)	Significant tightening of infraorbital laxity and skin. Surface area measurements also showed a decrease (6.31 ± 4.94 mm^2^ right eye, 4.78 ± 3.51 mm^2^ left eye), suggesting skin tightening
Park et al. (2015) [26]	Prospective clinical study	*N* = 20; Female = 90%; Ethnicity: Korean	Seven different facial areas	Single session of MFU‐V (4.5 and 3.0 mm depths)	All areas improved at 3 and 6 months, with significant gains at 3 months sustained. Jawline and periorbital areas had the most significant wrinkle and laxity reductions. Satisfaction scores of 3 or higher were reported at 3 and 6 months, particularly in the jawline, perioral, and cheek areas, and these levels lasted for at least 6 months
Rho and Chung (2018) [59]	Case Report	*N* = 1; Female = 0%; Ethnicity: Korean	Infraorbital area	Single session MFU‐V (1.5 and 3 mm depths) with submuscular PDO threads	Significant improvement in infraorbital laxity and wrinkles was observed 10 weeks after the combined treatment
Rokhsar et al. (2015) [60]	Prospective, open‐label pilot clinical trial	*N* = 20; Female = 100%; Ethnicity: Caucasian, Asian	Bilaterally above the elbows	Single session MFU‐V (4.5 and 3.0 mm depths)	Masked blinded assessment: 56% improvement at 90 days. SGAIS: 83% improvement at 90 days, 81% at 180 days. PGAIS: 94% improvement at both 90 and 180 days. Patient satisfaction: 83% noticed improvement at 90 days, 81% at 180 days
Sasaki et al. (2017) [14]	Randomized split‐face study	*N* = 20; Female = 90%; Ethnicity: Hispanic, Caucasian	Marionette folds	Single session Study 1: MFU‐V (4.5 and 3.0 mm) Study 2: MFU‐V (4.5 and 3.0 mm) versus 3 planes (adding 1.5 mm)	Delayed collagen remodeling contributes to softening of wrinkle lines. Patients showed improvement in lines and wrinkles of the décolleté
Shome et al. (2019) [61]	Prospective, double‐blind study	*N* = 50; Female = 52%; Ethnicity: Indian	Mid and lower face	Single session MFU‐V (3.0‐mm for forehead, temples, malar, and 4.5 and 3.0‐mm for cheeks and submental areas)	Improvements reported by 93% of blinded reviewers and 85% of patients at 6 months, maintained at 1 year
Smith et al. (2020) [62]	Prospective, pretreatment/posttreatment study	*N* = 60; Female = 100%; Ethnicity: Caucasian, Hispanic, African American, Asian	Bilateral outer thighs	Single session MFU‐V (3.0 and 4.5 mm depths) plus dilutes CaHA‐CMC	BODY‐Q Appearance Scales, Satisfaction Scales, Psychosocial Distress Scale 54% improvement in body image; 39% decrease in perception of excess skin (*p* < 0.01); significant gains in firmness
Suh et al. (2011) [5]	Clinical and histological evaluation	*N* = 22 (11 biopsies); Female = 91%; Ethnicity: Korean	Entire face	Single session MFU‐V (3.0 and 4.5 mm depth)	All patients' nasolabial folds and jawlines showed improvement, with 91% demonstrating a two‐score improvement and 9% one‐score improvement. 77% reported significant improvement in nasolabial folds, and 73% in the jawline. Histology: 23.7% increase in collagen (*p* = 0.001), and dermal thickness increased 65.9% (*p* = 0.001). Elastic fibers in the reticular dermis appeared straighter
Suh et al. (2012) [63]	Clinical study	*N* = 15; Female = 87%; Ethnicity: Asian	Lower eyelids	One or two treatments of MFU‐V (3.0 mm depth)	Objective: 13.33% much improved, 73.33% improved, 13.33% unchanged. Subjective: 20.00% much improved, 80.00% improved. Histology showed significant regeneration, increased dermal collagen and elastic fibers, and thickening reticular dermis
Vachiramon et al. (2020) [64]	Prospective, single‐blinded, randomized, controlled study	*N* = 28; Female = 100%; Ethnicity: n/s	Abdomen	Single‐plane MFU‐V(4.5 mm) versus Dual‐plane MFU‐V (4.5 mm then 3.0 mm depth). Single session	Similar physician‐evaluated scores at 3 and 6 months. No significant difference between protocols was observed. Patients post‐childbirth showed higher improvement scores for skin laxity
Vachiramon et al. (2021) [65]	Randomized, single‐blinded, controlled study	*N* = 27 Female = 93% Ethnicity: n/s	Middle part of the posterior upper arm	Single‐plane MFU‐V(4.5 mm) versus Dual‐plane MFU‐V (4.5 mm then 3.0 mm depth). Single session	Physician‐rated scores showed single‐plane treatment was superior to dual‐plane (*p* < 0.05). Patient‐rated scores were not statistically significant
Werschler and Werschler (2016) [66]	Prospective, open‐label pilot study	*N* = 20; Female = 85%; Ethnicity: Caucasian, Hispanic/Latino	Face and neck	Single session customized dual‐depth MFU‐V treatment was administered using depths of 1.5, 3.0, and 4.5 mm	PGAIS scores showed 100% improvement at 90 and 180 days, and 95% at 1 year. SGAIS scores showed 90% improvement at 90 days and 95% at 180 days and 1 year. Self‐reported improvements; less sagging (79%) for up to one year. 30%–40% reporting ≥ 1 mm lower face lift at 90 days. 31%–38% reporting ≥ 0.5 mm eyebrow lift
White et al. (2007) [10]	Ex vivo, cadaveric study	*N* = 6 cadaveric specimens; Female = 67%; Ethnicity: n/s	Multiple facial regions	MFU‐V	Reproducible TIZs in the SMAS caused focused thermal collagen denaturation, leading to tissue shrinkage. Higher energy and high‐density patterns increased shrinkage, with immediate collagen contraction. Greater energy resulted in more contraction
Wood et al. (2024) [67]	Single‐center, prospective, randomized, investigator‐blinded clinical trial	*N* = 41; Female = 100%; Ethnicity: FST II‐V	Lower face, upper neck	Single session MFU‐V (4.5 mm then 3.0 mm depth). Standard versus customized protocol	Both groups showed significant jawline laxity at 6 months (*p* < 0.01). 74% of standard group and 77% of custom group had ≥ 1‐point improvement at month 6
Yutskovskaya et al. (2020) [7]	Randomized, split‐face, comparative clinical study with immunohistochemical analysis	*N* = 19; Female = 100%; Ethnicity: n/s	Lower third of the face, neck, décolleté, and right lower abdomen	Diluted CaHA‐CMC at visits 1, 2, 4, 5. MFU‐V at visit 3	Improvements in marionette lines, jawline, neck; 3D imaging shows improvements in submental volume and cervicomandibular angle. Significant increase in collagen I and III, elastic fibers, and angiogenesis (*p* < 0.0001). High satisfaction on GAIS; remodeling lasted 15 months
Glow					
Casabona (2020) [29]	Retrospective study	*N* = 48; Female = *n*/s; Ethnicity: n/a	Lower face and neck	Single session MFU‐V (1.5, 3.0, and 4.5 mm); 30 days after thread insertion	Patient Satisfaction Scale rose at 90 days and 1 year (*p* < 0.001); 75% reported sustained results at 1 year. Perceptual shifts, though not direct radiance measures, highlight core glow components
Kerscher et al. (2019) [32]	Observational, single‐center, open‐label safety evaluation	*N* = 22; Female = 100%; Ethnicity: Caucasian	Lower face and submental region	Single session MFU‐V (4.5 and 3.0 mm depth)	Positive impacts on skin firmness potentially contributing to enhanced glow/radiance through improved skin structure
Lim (2023) [16]	Uncontrolled pilot study	*N* = 20; Female = 95%; Ethnicity: Chinese	Cheeks	Two sessions, 1 month apart. MFU‐V (1.5 mm depth)	Lightened melasma shade and decreased area; skin radiance not assessed but likely improved with lighter pigmentation. Sites with > 30% melasma involvement dropped from 55% to 20% by month 5
Smith et al. (2020) [62]	Prospective, pretreatment/posttreatment study	*N* = 60; Female = 100%; Ethnicity: Caucasian, Hispanic, African American, Asian	Bilateral outer thighs	Single session MFU‐V (3.0 and 4.5 mm depths) plus dilutes CaHA‐CMC	BODY‐Q Psychosocial Distress scale, 36% reduction in appearance‐related distress; overall perception of improved skin quality
Surface evenness					
Crepiness					
Cheng (2019) [47]	Case report	*N* = 1; Female = 100%; Ethnicity: n/s	Submental and submandibular areas	Single session MFU‐V (4.5‐ and 3‐mm) to submental/submandibular areas and lower cheek, with IncobotulinumtoxinA at lower middle mandible, platysma and to mouth corners	Noted improvement in skin texture, fine wrinkles, and dimpling at 1 and 3 months, contributing to smoother neck and chin contour
Jones et al. (2017) [53]	Randomized, evaluator‐blinded, comparative trial	*N* = 20; Female = 95%; Ethnicity: n/s	Neck	Single session of SMRF or MFU‐V	Improvements in texture and laxity/sagging skin implied a reduction in crepiness
Lee et al. (2012) [55]	Prospective Study	*N* = 10; Female = 100%; Ethnicity: FST III and IV	Face and neck	Single session MFU‐V (4.5 mm then 3.0‐mm depth)	Blinded evaluators rated 80% of patients as improved at 90 days, with 25% showing significant, 50% moderate, and 25% mild improvement. Patient assessments aligned with clinician ratings. Two‐pass protocol was effective in skin tightening and visible improvement in crepiness of the neck and lower face
Park et al. (2020) [35]	Pilot study	*N* = 20; Female = 100%; Ethnicity: Korean	Upper face	Single session MFU‐V (3.0 mm and 1.5 mm depth) on supra‐brow, lateral canthus, infraorbital areas; CPM‐HA fillers into sunken upper eyelid, lateral eyebrow, infraorbital areas; Incobotulinumtoxin‐A into forehead, glabella, crow's feet, lateral orbital rim	Both GAIS ratings increased at week 12 compared to immediately post‐treatment (*p* < 0.05), showing visible improvement in crepiness and upper facial texture
Sasaki and Tevez (2012) [68]	Clinical study	*N* = 82; Female = 99%; Ethnicity: Caucasian, Hispanic, Asian, African American	Periorbitum, knees, decollete, brachium, periumbilicus, inner thigh, hands, buttock	Single session MFU‐V 1.5 and 3.0 mm depths for periorbital skin, and 4.5, 3.0, and 1.5 mm levels for body sites	Moderate improvements in crepey skin around eyes, inner arms, belly button, knees, and décolletage lines. Significant reduction of gathered crepey lines above the knees
Smith et al. (2020) [62]	Prospective, pretreatment/posttreatment study	*N* = 60; Female = 100%; Ethnicity: Caucasian, Hispanic, African American, Asian	Bilateral outer thighs	Single session MFU‐V (3.0 and 4.5 mm depths); diluted CaHA‐CMC injected into subdermis	Satisfaction with Hips and Outer Thighs scale increased by 4.9 points (62%), with visible improvement in crepiness and contour related to wrinkles
Pore size					
Park et al. (2023) [69]	Single‐center, retrospective clinical study	*N* = 20; Female = 95%; Ethnicity: Asian	Anterior cheeks	Single session superficial MFU‐V and intradermal Incobotulinumtoxin‐A (INCO)	Mean pore count and density dropped significantly after 1 week, decreasing up to 62% until 24 weeks. Improvements lasted for 24 weeks with no rebound
Vachiramon et al. (2021) [70]	Randomized, single‐blinded, split‐face study	*N* = 46; Female = 70%; Ethnicity: Asian	Cheeks	Single session MFU‐V monotherapy (1.5 mm depth) or MFU‐V combined with HA filler injection	Mean pore volume significantly declined with both treatments, lowest at 4 months. Combined technique showed a nonsignificant lower pore volume. Physician pore grading also improved for both
Lee et al. (2015) [20]	Prospective randomized clinical trial	*N* = 22; Female = 86%; Ethnicity: Korean	Cheeks	Single session MFU‐V: one cheek with 1.5 mm transducer, the other with 3.0 mm	Objective clinical assessments showed pore improvements in 86% (1.5‐mm transducer) and 91% (3.0‐mm transducer) of sites. Approximately 45% of subjects reported satisfaction with pore improvements
Scars					
Casabona (2018) [71]	Nonrandomized, retrospective pilot study	*N* = 10; Female = *n*/s; Ethnicity: n/s	Face	Single session MFU‐V (3.0 and 1.5 mm depths) immediately followed by dilute CaHA‐CMC into the same areas	Significant improvement in baseline acne scar severity (*p* = 0.002). Notable improvements occurred in subjects with severe scars, as well as in hyperplastic papular scars
Casabona (2019) [44]	Retrospective, nonrandomized study	*N* = 20; Female = 100%; Ethnicity: n/s	Breasts, Buttocks, Thighs, Abdomen	Single session MFU‐V (3.0 and 1.5 mm) in a cross‐hatch pattern; subjects previously treated with CaHA, 20% ascorbic acid, and microneedling	Significant improvement in scar appearance at 90 days (*p* < 0.001)
Maas and Joseph (2019) [72]	Open‐label pilot study	*N* = 18; Female = 60%; Ethnicity: FST I‐V, Caucasian, Asian, Native American	Cheeks and/or temples	Triple session MFU‐V (3.0 and 1.5 mm), 30 days apart	Photograph assessment showed 100% scar improvement at 90 and 180 days. Investigator GAIS scores showed 100% of subjects were “Improved” or “Much Improved” at all time points. Subject GAIS scores showed nearly 90% continued improvement at 180 days
Wrinkles and lines					
Araco (2020) [40]	Prospective Study	*N* = 50; Female = 94%; Ethnicity: Caucasian, Asian	Face and neck	Single session MFU‐V (4.5, 3.0, and 1.5 mm depth)	Antera 3D analysis showed significant improvement in wrinkles
Barbarino (2021) [73]	Case series	*N* = 10; Female = 80%; Ethnicity: n/s	Periocular area and tear troughs	Single session MFU‐V (1.5 and 3.0 mm depths) followed by injection of CPM‐HA gel into tear troughs after 3 months	Describes synergy of structural tightening with volumization aiming to restore lower eyelid tautness and reduce sagging and wrinkles
Bartsch et al. (2020) [41]	Randomized interventional prospective study	*N* = 61; Female = 100%; Ethnicity: n/s	Gluteal and posterior thigh regions	Arm 1: TSGS (day 0) then MFU‐V (day 90). Arm 2: TSGS (day 0) then CaHA (day 90). Arm 3: TSGS (day 0) then MFU‐V + CaHA (day 90). Arm 4: MFU‐V + CaHA (day 0) then TSGS (day 90)	Significant improvement in dimpling across all treatments (*p* < 0.001). Triple modalities had higher odds of improvement than two modalities. Arm 4 showed the greatest improvement
Casabona and Nogueira Teixeira (2018) [74]	Retrospective study	*N* = 47; Female = 93.6%; Ethnicity: n/s	Neck and/or décolletage	Single session MFU‐V (3.0 and 1.5 mm depths), followed by subdermal diluted CaHA‐CMC	Neckline and Décolletage scores both significantly improved at 90 days (*p* < 0.001). 93% of patients were very satisfied or satisfied with neckline, 94% with décolletage (*p* < 0.001)
Casabona and Pereira (2017) [43]	Retrospective study	*N* = 20; Female = 100%; Ethnicity: n/s	Buttocks and upper thighs	Single session MFU‐V followed by subdermal diluted CaHA‐CMC	Significant improvement on the cellulite severity scale (*p* < 0.001) with a 4.5‐point mean score reduction (*p* < 0.001) after one MFU‐V/CaHA treatment, indicating fewer wrinkles
Corduff and Lowe (2023) [21]	Pilot case study	*N* = 10; Female = 90%; Ethnicity: n/s	Full‐face and upper neck	Multi‐session MFU‐V using the Hi5 protocol	Improvement in skin and fibromuscular ptosis, soft tissue laxity. Mean brow height increased by 1.7 mm, and maximum brow height by 1.8 mm
Doh et al. (2018) [30]	Retrospective case series	*N* = 10; Female = 100%; Ethnicity: Korean	Lower neck	Single session MFU‐V, CPM‐HA filler, Incobotulinumtoxin‐A, and CaHA‐CMC (1 patient)	Significant reduction in horizontal neck line scores (*p* = 0.007); all 10 patients showed clinical improvement
Fabi et al. (2013) [18]	Single‐center, prospective cohort study	*N* = 21; Female = 100%; Ethnicity: FST I and II	Décolletage	Single session MFU‐V treatment (4.5‐ and 3.0‐mm depths)	Rhytides improved over time (*p* < 0.0001). 46% of subjects showed wrinkle improvement at day 90, and 62% at day 180. Patient questionnaires reported improvement in lines/wrinkles by 83% at day 90 and 90% at day 180
Fabi et al. (2015) [49]	Prospective, open‐label, multicenter pilot clinical trial	*N* = 125; Female = 100%; Ethnicity: White, Hispanic/Latino, Other	Décolleté	Single session MFU‐V (4.5, 3.0, and 1.5 mm depths)	Wrinkles and lines of the décolleté improved: blinded assessments showed 69.9% at 90 days and 66.7% at 180 days; subject reports indicated 84.5% improvement at 90 days and 27.5% at 180 days
Fabi et al. (2020) [75]	Clinical Trial	*N* = 15; Female = 100%; Ethnicity: n/s	Décolleté	Single session MFU‐V (4.5, 3.0, and 1.5 mm depths)	Significant decrease in dynamic and at‐rest wrinkle scores (*p* < 0.01) at days 90, 180, and 360. Score dropped 23.3% by week 12 and remained significant through day 360. Wrinkle improvements appeared at days 180 and 360
Juhász et al. (2023) [31]	Prospective, open‐label, split‐body study	*N* = 20; Female = 100%; Ethnicity: Caucasian, Black	Lower anterior thigh and knee	Single session MFU‐V (dual‐depth 3.0, 1.5 mm; or triple‐depth: 4.5, 3.0, 1.5 mm) combined with diluted CaHA intradermally	3D‐imaging analysis showed baseline average wrinkle depth of 0.21 ± 0.04 mm. At 12 weeks, it improved to 0.20 ± 0.05 mm (*p* = 0.36)
Jung et al. (2016) [54]	Evaluator‐blinded, split‐face study	*N* = 20; Female = 90%; Ethnicity: Korean	Mid‐face region	MFU‐V versus HIFU: split face. Both, 4.5 mm depth	Qualitative assessments showed mild to moderate improvement in wrinkle and line appearance
Koza et al. (2024) [76]	Split‐faced, randomized evaluator‐blinded trial	*N* = 17 Female = *n*/s Ethnicity: n/s	Periorbital area	Dual session (6–8 weeks apart) MFU‐V versus fractional 1550 nm laser. Split‐face: one lower eyelid and lateral orbit complex treated	Periorbital wrinkles improved from baseline, with 60%–70% dermatologist‐rated improvement. No significant difference between laser and ultrasound
Kwon et al. (2018) [34]	Clinical evaluation	*N* = 22; Female = 100%; Ethnicity: Korean	Face	Single session MFU‐V session followed by 20% glucose injection in temporal areas	Improvements in fine wrinkles were prominent in 15 patients (67%). Nasolabial fold improvement was evident from 1 week
Lowe (2021) [23]	Single‐center, Prospective case series	*N* = 9; Female = 100%; Ethnicity: n/s	Periorbital, perioral, accordion lines	Single session MFU‐V treatment (1.5 mm)	Clinicians observed clear improvements in periorbital (6/6) and accordion lines (5/6) at 180 days, with lesser improvement in perioral lines (3/6). Self‐assessments aligned
Lu et al. (2017) [57]	Single‐site, prospective, nonrandomized clinical trial	*N* = 25; Female = 92%; Ethnicity: Asian	Face, neck	Single session MFU‐V lines (4.5 mm for cheeks/neck; 3.0 mm for forehead/temple/cheeks/neck/infraorbital)	Mean VISIA wrinkle score decreased significantly at 90 days (*p* = 0.0222) but not at 180 days
Park et al. (2015) [26]	Prospective clinical study	*N* = 20; Female = 90%; Ethnicity: Korean	Seven different facial areas	Single session MFU‐V (1.5 and 3.0 mm)	All seven facial areas improved at 3 and 6 months. Significant progress at 3 months that persisted at 6 months. The jawline and periorbital areas had the greatest wrinkle and laxity score reductions. Patient satisfaction scores persisted at least 6 months, especially in the jawline, perioral, and cheek areas
Suh et al. (2011) [5]	Clinical and histological evaluation	*N* = 22 (11 biopsies); Female = 91%; Ethnicity: Korean	Entire face	Single session MFU‐V (3.0 mm and 4.5 mm depth)	Objective: All patients improved in nasolabial folds and jaw lines. 91% improved two scores, 9% improved one. Subjective: 77% reported much improvement in nasolabial folds, 73% in jaw line. Histological: Collagen increased by 23.7% (*p* = 0.001), dermal thickness rose (65.9%, *p* = 0.001)
Suh et al. (2019) [6]	Clinical and histological evaluation	*N* = 10; Female = 100%; Ethnicity: Korean	Periorbital wrinkles	Single session MFU‐V (1.5 mm depth)	Significant improvement in periocular wrinkles. 40% of patients reporting excellent and 40% good results. Objective improvements were highest for fine wrinkles in the crow's feet area. Collagen increase most in the upper dermis (28.22%) and elastic fibers most in the lower dermis (33.04%)
Vachiramon et al. (2021) [65]	Randomized, single‐blinded, controlled study	*N* = 27; Female = 93%; Ethnicity: n/s	Middle part of the posterior upper arm	Single session MFU‐V (Single‐plane 4.5 mm depth vs. Dual‐plane 4.5 and 3.0 mm depths)	ASLSS wrinkle scores decreased at 1 and 3 months, returned toward baseline at 6 months, but overall remained lower
Werschler and Werschler (2016) [66]	Prospective, open‐label pilot study	*N* = 20; Female = Ethnicity: Caucasian, Hispanic/Latino	Face and neck	Single session customized dual‐depth MFU‐V treatment (1.5, 3.0, and 4.5 mm depths)	Self‐reported fewer lines and wrinkles (58%) for up to 1 year
Wood et al. (2024) [67]	Single‐center, prospective, randomized, investigator‐blinded clinical trial	*N* = 41; Female = 100%; Ethnicity: FST II‐V	Lower face, upper neck	Single session MFU‐V (4.5 mm then 3.0 mm depth). Standard versus customized protocol	Improvements in surface evenness were supported by investigator‐assessed GAIS scores, which were significantly better in the customized protocol group (*p* = 0.01) at 6 months
Woodward et al. (2014) [37]	Retrospective analysis	*N* = 100; Female = *n*/s; Ethnicity: n/s	Face and neck	Single session MFU‐V (4.5‐ or 3.0‐mm), plus Fractional CO_2_ laser or eCO2 laser	Significant improvement in rhytides and photodamage texture. Combination addressed both superficial and deep layers for comprehensive aesthetic improvement
Tone evenness					
Coloration					
Lim (2023) [16]	Uncontrolled pilot study	*N* = 20; Female = 95%; Ethnicity: Chinese	Cheeks	Two sessions, 1 month apart. MFU‐V (1.5 mm depth)	Melasma visible improvements in 20% of cheeks occurred as early as 1 month post‐treatment. Surface area reduction was seen in 40% of sites after the second treatment and 70% by month 5
Erythema					
Schlessinger et al. (2019) [17]	Pilot study	*N* = 91; Female = 90%; Ethnicity: White, non‐Hispanic	Central face	Single high‐density or two low‐density MFU‐V treatments (4.5, 3.0, and 1.5 mm)	Mean colorimetry showed modest improvements in erythema: success rates ranged from 56.5% to 75% at day 90, 68.1%–73.7% at day 180, and 71.4%–86.4% at day 365
Pigmentation					
Chan et al. (2011) [15]	Safety study, prospective, clinical trial	*N* = 49; Female = 92%; Ethnicity: Chinese	Full‐face	One‐to‐three session MFU‐V (3.0 and 4.5 mm depths) spaced 4 weeks apart	Two cases of post‐inflammatory hyperpigmentation (PIH) appeared on the forehead after 1 month, resolving by 6 months. It was linked to the deeper 4.5 mm transducer and resolved with a more superficial transducer
Lim (2023) [16]	Uncontrolled pilot study	*N* = 20; Female = 95%; Ethnicity: Chinese	Cheeks	Two session MFU‐V (1.5 mm depth) treatments spaced 1 month apart	The mean mMASI score dropped significantly at month 4 and 5. 72.5% of cheeks showed melasma lightening at month 4, with 75% improvement through month 5. 40% of sites improved 1 month after treatment, with improvements after the second treatment until study end
Vachiramon et al. (2018) [25]	Randomized, evaluator‐blinded pilot study	*N* = 20; Female = 60%; Ethnicity: Asian	Left arm, UVB‐induced pigmentation	Single session MFU‐V (1.5 mm depth)	Control sites Lightness Index (L) improved from 36.76 to 37.77; MFU‐V sites from 35.80 to 36.76 at 4 weeks. Blinded dermatologists graded improvement scores: Fitzpatrick III, controls improved more than MFU‐V. For Fitzpatrick IV, MFU‐V sites improved more. Histology showed greater melanin reduction in sites for Fitzpatrick III and IV

### 2.1 Skin Firmness

Skin firmness is defined by elasticity, tautness/tightness, and hydration—all of which contribute to the skin's viscoelastic properties conditioned by the underlying tissues [1]. Numerous studies have demonstrated the potential of MFU‐V to restore skin physiological function and firmness. Elasticity improvements have been documented following both single and multiple MFU‐V treatments [14, 20, 21].

Kerscher et al. observed significant increases in net elasticity compared to baseline at 12 and 24 weeks (*p* < 0.05) [32]. Suh et al. reported a visible improvement in nasolabial folds and jawline in 91% of participants 2 months after MFU‐V treatment. This was corroborated by histological evidence of a thicker dermis, increased dermal collagen, and reorganization of elastic fibers [5].

**FIGURE 3:**
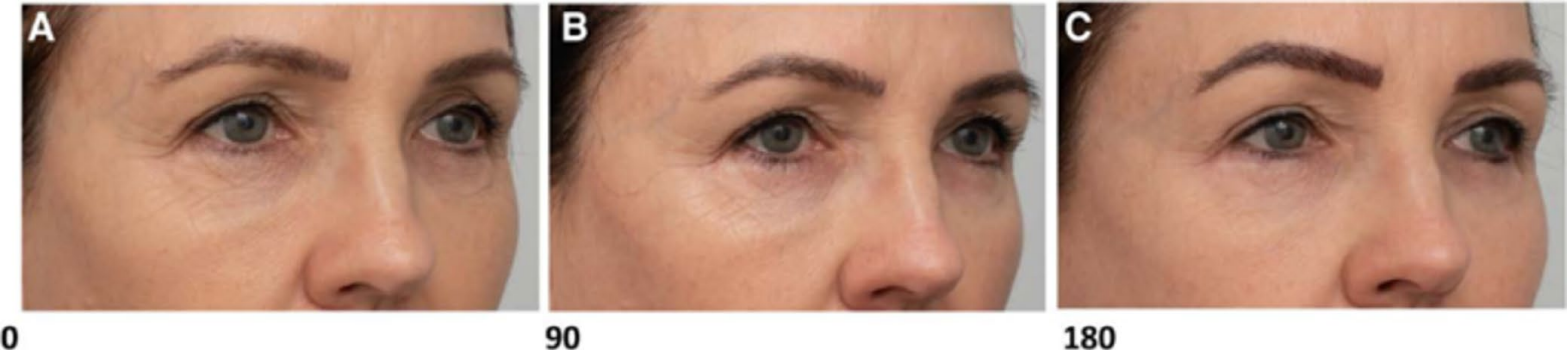
Periorbital lines. (A) At baseline 0. (B) At 90. (C) At 180 days. Assessor rated this case as “much improved” at day 90 and “exceptionally improved” at day 180. Figure reproduced from: Lowe, *Plast Reconstr Surg Glob Open*. 2021; 9:e3662. doi:10.1097/GOX.0000000000003662. Licensed under CC BY‐NC‐ND 4.0.

A randomized split‐face follow‐up study by Sasaki et al. examined the treatment of marionette lines with MFU‐V. Significant improvements in photographic tissue displacement and Cortex elasticity were shown at 180 and 360 days when three focal depths were treated versus two, suggesting a depth‐dependent benefit [14]. Corduff and Lowe's study provides an alternative insight into the standard MFU‐V approach by evaluating the effects of a single high‐density treatment session using a 10‐MHz/1.5 mm transducer, with follow‐up conducted at 6 or 10 months. Their findings demonstrated a mean brow lift of 1.7 mm, improved ptosis, and a submental lift averaging 78.7 mm, indicating improved skin elasticity and tightness. Furthermore, physician‐assessed Global Aesthetics Improvement Scores (GAIS) showed clinical improvement in 87% of patients. The Scientific Assessment Scale of Skin Quality (SASSQ) also revealed reduced wrinkle severity and skin roughness (Figure 3) [21]. These results suggest that intensified, focused treatment sessions may yield efficient outcomes in structural support and visible skin texture improvement.

Additional studies support the benefits of MFU‐V combination treatments on skin elasticity across facial and body regions. Kerscher et al. demonstrated improvements in submental tightening and density, with sustained dermal thickening observed through 48 weeks in the lower face after the application of calcium hydroxylapatite (CaHA), administered 12 weeks post‐MFU‐V treatment if results from the energy‐based device were not satisfactory [33]. In the upper arms, Ramirez et al. reported significant quantitative cytometry gains in both firmness (*p* < 0.05 at 12 and 24 weeks) and elasticity (*p* < 0.05 at 4, 12, and 24 weeks) after a single session of MFU‐V and diluted CaHA, with 88% of patients showing aesthetic improvement [36].

Tightening has been consistently reported across various treatment areas, including the abdomen [56, 64], the upper arm and the elbow [39, 60, 65], the knees [39, 51], buttocks [52], décolletage [18, 49], lower eyelids [58, 63], the mid and lower face [5, 20, 26, 40, 46, 54, 57, 61], lower face and neck [19, 22, 32, 38, 42, 45, 48, 49, 50, 55, 57, 67] marionette lines [14], and the periorbital region and accordion lines [23]. Systematic review and meta‐analyses substantiate positive tightening effects of MFU‐V. Contini et al. reported brow lift improvements of 0.47–1.70 mm and submental reductions of 25–45 mm^2^, with investigator‐rated skin tightening in 92% of cases at 90 days post‐treatment, and patient‐reported satisfaction increasing from 42% to 53% over 1 year [77]. Ling and Zhao's meta‐analysis of facial tightening and rejuvenation studies showed a response rate of 77% (95% CI: 58%, 96%) at 90 days and 69% (95% CI: 51%, 87%) at 180 days post‐treatment, with patient satisfaction rates over 70% [78].

Building on a wealth of tightening evidence for MFU‐V alone, several studies have explored the additional effects of combination treatments involving MFU‐V and other modalities. Bartsch et al. demonstrated that pairing MFU‐V with diluted CaHA, followed by tissue‐stabilized‐guided subcision 3 months later, yielded the highest odds of a ≥ 1‐grade improvement in gluteal and thigh skin laxity (OR: 2.23, 95% CI 0.51–9.82) at 9 months [41]. Similarly, Casabona reported significant improvements in striae‐associated tautness following MFU‐V in areas previously treated with CaHA, microneedling, and ascorbic acid, with Manchester Scar Scale scores improving from 9.35 ± 1.18 to 6.30 ± 1.26 (*p* < 0.001), supported by histological evidence of dermal remodeling [44].

In the outer thighs, Smith et al. combined MFU‐V and diluted CaHA, resulting in a 54% improvement in body image and a 39% reduction in perception of excess skin (*p* < 0.01), along with significant gains in perceived firmness [62]. Casabona and Pereira found similar benefits in moderate‐to‐severe cellulite of the buttocks and thighs, with a single MFU‐V and diluted CaHA session producing significant improvement on the cellulite severity scale (*p* < 0.001) and in tissue firmness and skin tightness [43].

Histological studies lend further weight to the combination treatment findings. In a single facelift skin sample, Casabona et al. observed a threefold increase in dermal and epidermal thickness and improvements in the 3D dermal matrix when MFU‐V, CaHA, cohesive polydensified matrix‐hyaluronic acid (CPM‐HA, Belotero, Anteis S.A., Plan‐les‐Ouates, Switzerland, a company of the Merz Aesthetics group) filler, and microneedling were combined, particularly when MFU‐V preceded filler [28]. Woodward et al. also reported that MFU‐V, combined with ablative laser resurfacing, yielded multi‐layer rejuvenation, enhancing both structural and surface‐level outcomes [37]. Additionally, Casabona and Marchese showed improved aging scores and maintained neocollagenesis in patients receiving MFU‐V 1 month after thread lifting, with effects lasting up to 1 year [29].

Clinical improvements in laxity, tautness, and contour were also seen in other anatomical regions. Cheng combined MFU‐V to the submentum and jawline with incobotulinumtoxinA injections to the platysma, mentalis, and depressor anguli oris, achieving improved cervicomental contour, chin projection, and lower face tautness sustained at 3 months [47]. Park et al. similarly reported eyebrow lifting and periorbital firming at 1, 4, and 12 weeks through a tri‐modal approach using MFU‐V, hyaluronic acid (HA), and botulinum toxin [35]. Kwon et al. treated midface laxity using MFU‐V and glucose injections, reporting 91% improvement at 12 weeks alongside histological increases in dermal collagen [34]. In the knee and thighs, Juhász et al. demonstrated significant gains in the Merz Skin Laxity Scale at 12 and 24 weeks with MFU‐V and diluted CaHA, supporting visible improvements in tautness [31]. Finally, Doh et al. combined MFU‐V, HA, CaHA, and incobotulinumtoxinA to treat neck laxity, achieving a significant reduction in laxity scores (from 1.30 to 0.725, *p* = 0.008) across all 10 patients, reinforcing the role of multimodal strategies in enhancing tissue tightness [30]. Collectively, these findings highlight the expanding role of MFU‐V as a foundational platform in multimodal protocols to address skin tightness across diverse body areas.

Results from a study by Kerscher et al. into the effects of MFU‐V on hydration and barrier integrity found that hydration levels remained stable post‐treatment, with no significant change in trans‐epidermal water loss at short‐ and long‐term measurements. Although there was a statistically significant reduction in hydration, as measured by corneometry, over 12 weeks (*p* = 0.0001), the measurements remained within typical physiological ranges, indicating no compromised skin function. These results support the conclusion that MFU‐V can enhance skin firmness while maintaining epidermal integrity and moisture balance [32].

### 2.2 Skin Surface Evenness

The second EPC, skin surface evenness, encompasses pore size, crepiness, wrinkles and lines, scarring, hair, and overall clarity. Improvements in this domain are often visibly apparent and play a key role in the perception of smooth, healthy‐looking skin. Because MFU‐V treatments improve skin, particularly through dermal remodeling and increased firmness, visual improvements in wrinkles and texture have been consistently reported across facial and upper body areas. Systematic reviews by Contini et al. and Ling and Zhao confirm significant gains in facial skin tightening and wrinkle reduction, reinforcing MFU‐V's role in improving both tactile quality and visible surface texture [77, 78]. By minimizing surface‐level irregularities, MFU‐V also supports more uniform light reflection, contributing to a smoother and more radiant skin appearance.

Multiple studies have demonstrated MFU‐V's effectiveness in refining texture and reducing pore size. In a study of Asian patients, Lee et al. reported significant reductions in visible pore size, as well as improved elasticity and surface evenness [20]. Complementing these findings, Park et al. implemented a customized MFU‐V protocol for enlarged pores using a 10‐MHz/1.5 mm transducer (100 lines per cheek), resulting in statistically significant reductions in pore number and size at 3 months post‐treatment (*p* = 0.018 and *p* = 0.017). A two‐grade improvement in pore grading and marked refinement of skin texture were observed, with physician and patient GAIS scores rated as “very much improved” [79].

The combination of MFU‐V with injectable agents has demonstrated benefits in reducing pore size. Park et al. achieved a reduction of up to 62% in pore count and density, as marked over 24 weeks, using MFU‐V and intradermal incobotulinumtoxinA [69]. Vachiramon et al. found positive findings in a split‐face study comparing MFU‐V alone to MFU‐V plus HA. While both arms showed a decline in pore volume, satisfaction rates and pore metrics were significantly better in the combination group [70]. These results reinforce the benefits of adjunctive therapies in achieving optimal skin texture outcomes.

**FIGURE 4:**
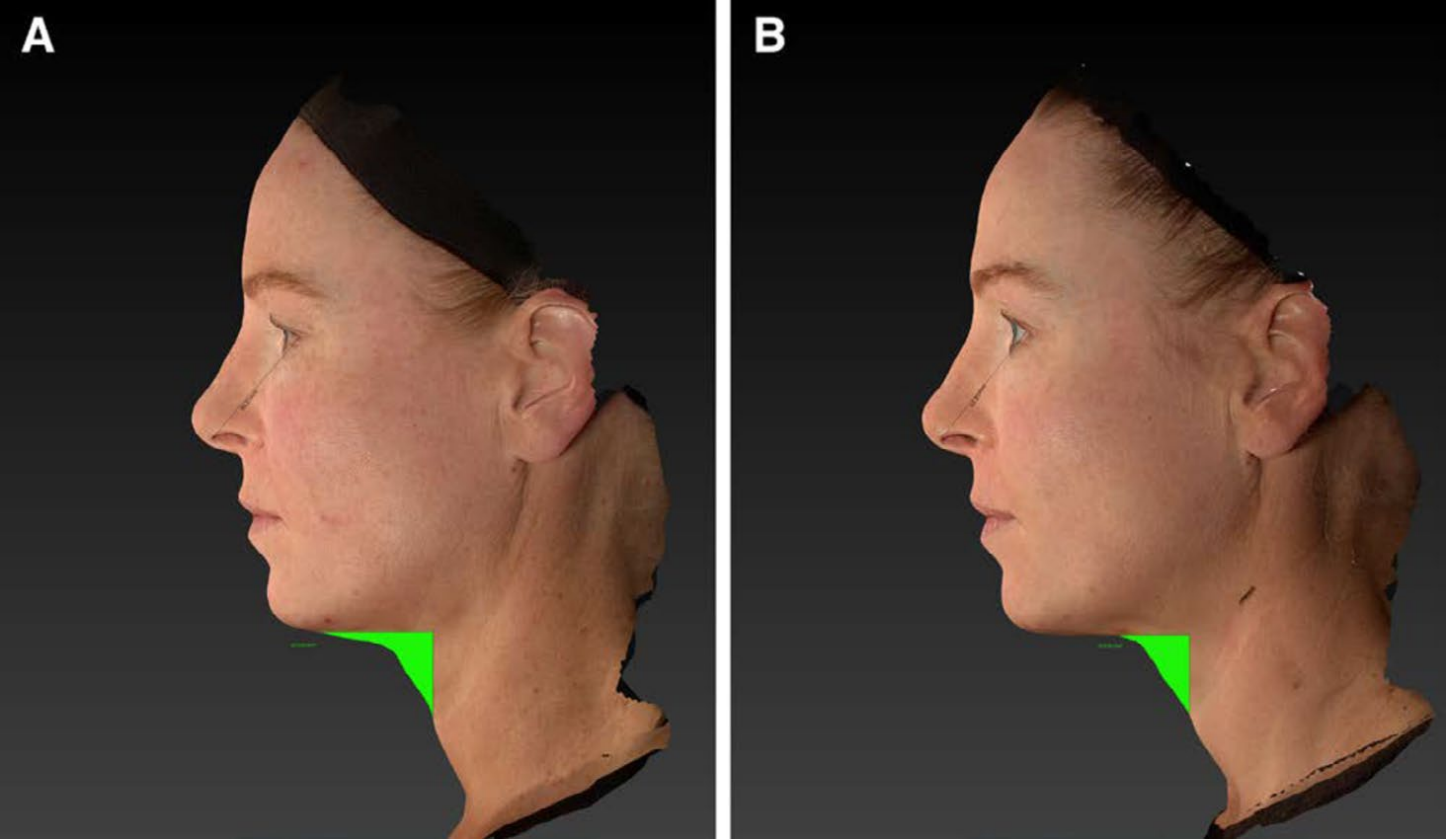
Measurement of changes in submental areas. Pretreatment (A) to posttreatment (B) changes are spatially indicated in areas highlighted in green. Figure reproduced from: Corduff and Lowe, *Plast Reconstr Glob Open*. 2023; 11:e5184. doi:10.1097/GOX.0000000000005184. Licensed under CC BY‐NC‐ND 4.0.

Several studies support the impact of MFU‐V on wrinkles and fine lines. A prospective case series by Lowe used a single 10‐MHz/1.5 mm transducer in nine women with superficial rhytids, reporting visible improvements in periorbital, accordion, and perioral lines at 180 days, with high satisfaction and tolerability (Figure 4) [23]. Similarly, Suh et al. treated 10 Korean women (Fitzpatrick III–IV) with a single 10‐MHz/1.5 mm transducer, achieving the highest objective improvement scores in crow's feet wrinkles (*p* = 0.043). Biopsy data revealed collagen increases in the upper dermis (+28.2%) and elastic fiber increases in the lower dermis (+33.0%), highlighting MFU‐V's capacity for remodeling thin, delicate skin areas [6].

Koza et al. further confirmed the efficacy of MFU‐V in periorbital wrinkle treatment through a randomized split‐face trial comparing it to a 1550‐nm fractional laser. MFU‐V achieved a 63.2% improvement at 3 months, with lower pain scores during the second session (*p* = 0.05), reinforcing its utility for wrinkle reduction with high tolerability [76]. In a prospective study of 20 Korean patients, Park et al. demonstrated significant improvement in physician evaluation scores for wrinkles across seven facial areas following a single MFU‐V session. Improvements were most pronounced in the jawline, cheek, and perioral regions, with high patient satisfaction and a favorable safety profile [26].

Prospective trials evaluating MFU‐V for facial and neck rejuvenation show qualitative improvements in wrinkle severity and surface texture after a single session. Dual‐depth treatment protocols highlight MFU‐V's customizability, leading to improvements in tightening and wrinkle reduction, as confirmed by the physician and subject GAIS scores [40, 66]. In a study of Taiwanese adults, Lu et al. demonstrated significant improvements in facial wrinkles at 90 days based on VISIA scores (*p* = 0.0222), alongside 84%–88% GAIS‐reported improvement at both 90 and 180 days. Gains were especially pronounced in the submental and brow regions, supporting the efficacy of MFU‐V in lifting and refining the visible surface [57].

In the décolletage, Fabi et al. reported significant reductions in dynamic and resting rhytids at 90, 180, and 360 days post‐treatment using 1.5, 3.0, and 4.5 mm transducers (*p* < 0.01), with patient satisfaction increasing over time [75]. Similarly, Gold et al., using a 4 MHz, 4.5 mm transducer followed by a 7 MHz, 3.0 mm transducer, found that 50% of patients reported smoother skin and fewer lines after MFU‐V treatment above the knees, with blinded review confirming improvements at both 90 and 180 days [51]. Werschler et al. observed that 95% of patients retained aesthetic improvements at 12 months using a dual‐depth, high‐density protocol with vertical vectoring. Among them, 58% reported fewer wrinkles and 47% noted a smoother texture, emphasizing the value of individualized delivery in anatomical regions such as the décolletage and neck [66].

Fabi's earlier work also demonstrated efficacy in the décolleté, with dual treatments using 3.0 mm and 4.5 mm transducers and 270 lines per session. By day 180, 62% of participants had achieved a two‐point improvement or less on validated wrinkle grading scales [18]. While Araco focused on face and neck rejuvenation, the study reported meaningful wrinkle and texture improvements using Antera 3D imaging for biomechanical analysis at 6 months following a single MFU‐V session with 1.5, 3.0, and 4.5 mm transducers. Significant 3D‐measured gains and high patient satisfaction support MFU‐V's multilayer treatment approach [40].

Several combination studies support MFU‐V's utility in reducing wrinkles and lines. Doh et al. observed significant horizontal neckline improvement (pre‐ to post‐treatment, 1.525 ± 0.257 to 0.75 ± 0.154, *p* = 0.007) following a single‐session combination of MFU‐V, CaHA, HA filler, and incobotulinumtoxinA [30]. Similarly, Casabona and Nogueira Teixeira reported significant improvement in wrinkles across the neckline and décolletage (*p* < 0.001), with a 93%–94% satisfaction rate [74]. Finally, Woodward et al. studied the use of MFU‐V and an ablative CO_2_ laser, targeting both deep and superficial skin layers, and documented improvements in skin laxity and rhytides. The authors concluded that the combination did not seem to increase recovery time or adverse event incidence [37]. In a retrospective chart review, Fabi et al. found no increase in adverse events when MFU‐V was used in combination with injectables, thereby reinforcing its safety in combined treatments [80]. These studies highlight the multifaceted effects of MFU‐V when combined with other modalities, particularly for wrinkle smoothing and texture refinement.

MFU‐V also improves skin evenness in scarred skin. Maas and Joseph treated 18 patients with moderate to severe atrophic acne scars using three sessions spaced 30 days apart with 10‐MHz/1.5 mm and 7‐MHz/3.0 mm transducers. Blinded reviewers rated 100% of patients as improved at 90 and 180 days, with most achieving 25%–50% improvement on the Acne Scar Improvement Scale (ASIS). Subject and investigator ratings aligned, underscoring MFU‐V's effectiveness for dermal remodeling in acne‐scarred cheeks and temples [72]. In a more recent study, Wood et al. evaluated MFU‐V's impact on surface quality using a randomized, investigator‐blinded protocol comparing standard dual‐depth treatment (4.5 and 3.0 mm transducers) with a customized protocol tailored to individual anatomy at the lower face and upper neck. Improvements in surface evenness were supported by investigator‐assessed GAIS scores, which were significantly better in the customized protocol group (*p* = 0.01), suggesting that individualized depth selection may enhance skin textural refinement alongside structural lifting. Utilizing multimodal therapies, Casabona and Marchese showed that priming of the scarred area with CaHA, microneedling, and ascorbic acid followed by MFU‐V produced significant improvements in striae scar quality, with a drop in Manchester Scar Scale scores and over 70% of patients reporting high satisfaction [81]. In acne scarring, Casabona demonstrated the benefit of dual‐depth MFU‐V with CaHA, with acne scar severity significantly improved, with the greatest improvement in subjects with severe scarring [71]. These findings suggest that MFU‐V's dermal remodeling capacity can be meaningfully enhanced through combination protocols for both atrophic and linear scars.

Jones et al. compared MFU‐V with subsurface monopolar radiofrequency (SMRF) in 20 patients with moderate neck laxity, including self‐assessed measures of texture and crepiness as secondary outcomes. MFU‐V patients reported a significant improvement in texture and crepiness, with physician‐ and patient‐reported outcomes showing visible smoothing by Day 30 (*p* = 0.0004), sustained through Day 180. In contrast, SMRF‐treated patients only reached comparable improvement levels by Day 180 (*p* = 0.0017) [53]. Sasaki and Tevez further support MFU‐V's application in reducing perceived crepiness across multiple body regions. In a prospective trial evaluating periorbital, décolleté, upper arm, periumbilical, knee, hand, and buttock regions, physician‐assessed improvements in crepiness and fine wrinkles were reported in all areas, most noticeably in the periorbital, décolleté, upper arm, knee, and periumbilical regions [68]. These findings suggest that MFU‐V improves skin firmness and crepiness, effectively remodeling the superficial dermis to reduce the appearance of fine, crinkled skin, particularly in delicate areas such as the neck.

Combination protocols involving MFU‐V have demonstrated meaningful improvements in crepiness, particularly in delicate or lax skin regions. Smith et al. showed a 4.9‐point improvement in satisfaction scores for hips and outer thighs following MFU‐V plus CaHA, noting visible contour and skin smoothness refinement [62]. At the same time, Cheng reported improved texture and fine wrinkles in the submental and chin region post MFU‐V and Xeomin [47]. In the periocular region, Barbarino demonstrated that MFU‐V, followed by HA filler at 3 months, visibly improved under‐eye crepiness via fine wrinkling reduction and tear trough contour, with all patients self‐rating as “very much improved” after combination treatment [73]. Park et al. reported significant improvements following a combined MFU‐V, HA, and botulinum toxin protocol. Average and maximum eyebrow height were measured significantly higher by the 12‐week assessment period (*p* < 0.05), with corresponding physician‐assessed visual analog scores (VAS) for the periorbital area, eyebrow ptosis, and infraorbital hollow all showing continuous reductions in severity. Both physician and patient GAIS ratings were significantly elevated compared to ratings immediately post‐treatment at week 12 (*p* < 0.05), reinforcing the visible improvement in crepiness and upper facial texture with this multimodal approach [35]. Adding to the multimodal applicability, Rho and Chung published a case study of a 10 MHz/1.5 mm and 7 MHz/3 mm transducer MFU‐V treatment followed by absorbable polydioxanone threads in the infraorbital region, 2 months post‐blepharoplasty. The patient showed visible improvements in mid‐cheek laxity, under‐eye fine lines, and static creases 10 weeks after the non‐surgical treatment [59]. These findings collectively highlight MFU‐V's capacity to enhance skin texture, particularly in delicate anatomical areas and when paired with HA or neuromodulators, or other techniques in treatments tailored to thin, finely wrinkled skin.

### 2.3 Skin Tone Evenness

Skin tone evenness is defined by three main attributes: pigmentation, coloration, and erythema [1]. While coloration and pigmentation contribute to the skin's overall appearance, pigmentation is primarily linked to melanin distribution and the skin's response to ultraviolet (UV) radiation. Conversely, coloration is influenced by various factors, including blood flow, pigments such as carotenoids, and external elements like sun exposure. Hyperpigmentation generally refers to the skin's darkening caused by various factors, including sun exposure, inflammation, and specific diseases or medications. Age‐related pigmentation disorders may also be influenced by the accumulation of senescent fibroblasts, which suppress stromal cell‐derived factor 1 (SDF‐1) and contribute to increased melanogenesis [82]. Erythema refers to the redness caused by vascular dilation or inflammation. Uneven tone, manifesting as hyperpigmentation, melasma, or erythema, can significantly affect skin quality and age perception. While MFU‐V does not directly target melanin, its ability to deliver energy below the epidermis enables it to remodel tissue and influence tone‐related features, such as vascular appearance or dermal support, while maintaining a strong safety profile across all skin types.

Translating to humans, Vachiramon et al. conducted a prospective, evaluator‐blinded trial in individuals with UVB‐induced hyperpigmentation on the arm. Their results demonstrated that MFU‐V treated sites in Fitzpatrick IV subjects showed significant improvement in mean lightness index scores (mean score 1.67 vs. 0.83, *p* = 0.049). Conversely, Fitzpatrick III patients achieved a greater mean lightness index in control sites than treated sites at 4‐week follow‐up (40.50 and 38.43, respectively, *p* = 0.160). At the 4‐week follow‐up, one of the two Fitzpatrick III patients and the sole Fitzpatrick IV patient sampled showed a greater reduction in histologically viewed melanin than the control group [25].

This therapeutic benefit may translate clinically into the treatment of melasma. In a pilot study by Lim et al., 20 Asian patients with mixed melasma received two sessions of superficial MFU‐V using a 10‐MHz, 1.5 mm transducer, spaced 1 month apart. The mean Melasma Area and Severity Index (MASI) score decreased significantly from 13.2 at baseline to 2.4 by month four, with 72.5% of cheeks visibly lighter. The results persisted through month five, and patient satisfaction was high throughout the study. These findings suggest that MFU‐V can provide consistent melanin reduction in affected areas, but does so safely and comfortably for patients with Fitzpatrick skin types III and IV [16].

Safety data from Chan et al. further underscore MFU‐V's suitability for pigment‐rich skin. In a cohort of 49 Chinese patients undergoing facial skin tightening, only two instances of PIH were reported, both of which were localized to the forehead and fully resolved within 9 months. No other cases of PIH were seen in the study after switching from the 7.0 MHz/4.5 mm transducer to the 7.0 MHz/3.0 mm transducer for treatments of the forehead region. While rare, these events highlight the importance of real‐time visualization, appropriate transducer selection, and controlled energy delivery, particularly when treating melanin‐sensitive regions [15].

MFU‐V has also shown promise in improving vascular‐related irregularities that contribute to uneven skin tone. In a 12‐month pilot study of 91 adults with erythematotelangiectatic rosacea, Schlessinger et al. treated participants with one or two MFU‐V treatments at low or high density. By 90 days, 75%–91.3% had achieved at least a one‐point improvement in Clinician Erythema Assessment (CEA) scores, the study's primary endpoint. Notably, results were durable through 180 and 365 days. Despite the inflammatory nature of the patient population, erythema as a side effect was reported in only 35% of patients [17].

Transient erythema is also one of the most consistently reported side effects across MFU‐V studies [10, 11, 15, 16, 17, 20, 21, 22, 23, 24, 25, 26]. It is typically mild, expected, and resolves without intervention. In a prospective study, Kerscher et al. evaluated erythema using Mexameter units (MU), observing a progressive decline over 12 weeks post‐treatment, from a baseline mean of 313.7–290.3 MU, following a single MFU‐V session using 4.5 and 3.0 mm transducers (800–900 lines) to the lower face and submental region, supporting erythemal resolution without post‐treatment intervention [32]. Its frequency across studies underscores the importance of patient education and proper post‐treatment care, rather than concern for long‐term complications or effects on skin quality. Real‐time visualization is key in minimizing risk by ensuring accurate coupling and controlled energy delivery, which is especially important when treating melanin‐rich or vascular areas.

Preclinical and clinical data support MFU‐V as a safe and effective modality for improving skin tone evenness. By addressing pigmentary disorders such as melasma and post‐inflammatory hyperpigmentation, and reducing vascular redness, MFU‐V offers a multifaceted approach to skin rejuvenation. Its ability to deliver targeted thermal stimulation without compromising the epidermis makes it well‐suited for diverse skin types and conditions where tone irregularity is a primary concern.

### 2.4 Skin Glow

Skin glow refers to the visible radiance and luminosity of the skin, a perceptual outcome tied to overall skin health. While not a discrete biological marker, glow reflects a combination of epidermal clarity, dermal integrity, smooth surface texture, and evenly reflected light. The attributes are strongly influenced by pigmentation uniformity, skin hydration, collagen structure, and microvascular tone. Dullness, or lack of skin glow, is often the result of uneven tone, surface roughness, enlarged pores, or reduced firmness, all of which cause light to scatter irregularly and diminish luminosity.

MFU‐V's impact on these underlying factors is well‐documented. As detailed in previous sections, the device improves pigmentation, erythema, collagen structure, and skin texture. These changes collectively enhance the skin's ability to reflect light more evenly.

While no current studies determine the effects of MFU‐V on perceived “glow,” some findings align with the perceptual components it represents. In a prospective trial of MFU‐V for melasma, Lim reported significant reductions in pigment density and lesion area, accompanied by steadily improving GAIS scores and high patient satisfaction over 5 months [16]. These outcomes reflect enhanced visual uniformity and overall skin appearance, without specific reference to brightness or luminosity. Similarly, Kerscher et al. observed significant improvements in skin elasticity and maintained epidermal barrier function at 12 and 24 weeks post‐treatment, without changes in erythema or hydration [32]. These physiologic improvements contribute to smoother, more resilient skin characteristics often linked to a more vibrant appearance.

Complementing these effects, MFU‐V combination strategies have also shown promise. Smith et al. reported a 36% reduction in appearance‐related psychosocial distress following a single session of MFU‐V with CaHA in women with thigh laxity, suggesting improved skin confidence and perceived quality [62]. Casabona found that combining MFU‐V with thread lifting significantly increased patient satisfaction, from 3.21 to 4.46 at 90 days, maintaining at 4.29 at 1 year. These perceptual shifts, though not direct measurements of radiance, highlight improvements in skin smoothness, firmness, and structural harmony, core components of the glow experience [29]. While combination approaches may enhance patient satisfaction and perceived outcomes, recent studies highlight the importance of appropriate sequencing for safety and efficacy. Harnchoowong et al. found that MFU‐V applied within 14 days of intradermal incobotulinumtoxinA significantly reduced its anhidrotic effect, recommending a minimum 2‐week delay to preserve neurotoxin function [83]. Similarly, Vachiramon et al. observed that applying MFU‐V within 14 days of HA filler injection led to reduced filler persistence, whereas delaying MFU‐V by 28 days preserved dermal filler integrity [84]. These findings underscore the need for procedural planning in multimodal treatment protocols to avoid compromising clinical outcomes.

## 3 Conclusion

This review identified 70 studies evaluating the effects of MFU‐V on skin quality, spanning clinical, histological, preclinical, and patient‐reported outcomes. Evidence was strong for improvements in skin firmness, reported in 52 studies across diverse anatomical regions, including the lower face, submental area, neck, thighs, and arms. Improvements were frequently documented using physician‐assessed grading, blind evaluator scores, histological analyses, and validated imaging tools. Follow‐up durations ranged widely, with the majority of studies assessing outcomes at 90 days, 180 days, and seven studies extending to 12 months or beyond [7, 14, 17, 41, 66, 73, 75], indicating both short‐term visible lifting and longer‐term remodeling effects.

Surface evenness was assessed in 35 studies, with improvements noted in parameters such as texture, rhytides, and pores, particularly in facial and periorbital regions. Tone evenness and glow were evaluated less frequently (4 studies each), with measurement tools varying widely. Indeed, direct quantification of glow has been limited up to now and may be inferred from indirect improvements in tone, texture, and smoothness. The growing use of validated imaging methods (e.g., EI, MI, gloss metrics) supports increasing objectivity in these domains [85].

While most studies in this review evaluated single‐session protocols, a smaller subset explored multiple MFU‐V treatments, particularly in areas such as scarring [72] and melasma [16]. Multiple sessions were also found to affect skin elasticity, as well as patient comfort and affordability through staggered stages of treatment [21]. Furthermore, treatment was not linked to PIH adverse events; instead, it was attributed to deeper‐layer treatment [15]. These cases suggest a potential incremental benefit with repeated sessions in selected patients. While subjective and objective improvements in skin quality were generally maintained or enhanced over time [16, 21, 76], direct comparisons to single‐session treatments remain limited.

MFU‐V exhibits a favorable safety and tolerability profile, with transient erythema, edema, and discomfort being the most commonly reported adverse events [10, 11, 15, 16, 17, 20, 21, 22, 23, 24, 25, 26, 32]. Its use across a wide range of Fitzpatrick skin types and anatomical regions, including face, neck, submental area, and off‐label body sites, supports its versatility in aesthetic practice. Combination treatments with fillers or neurotoxins were also reported on 19 occasions, highlighting MFU‐V's compatibility within multimodal strategies [1, 2, 3, 4, 5, 6, 7, 8, 9, 10, 11, 12, 13, 14, 15, 16, 17, 18, 19].

While skin quality is not a regulatory treatment indication, the accumulated evidence suggests that MFU‐V contributes meaningfully to quantifiable and perceptible improvements across skin quality domains. The integration of biophysical measures, perceptual assessments, and patient‐reported outcomes, along with standardized frameworks such as the EPC model, will be critical to advancing research and guiding individualized care. Ongoing refinement of protocols, particularly in terms of session frequency, treatment depth, and combination strategies, will help optimize both clinical outcomes and patient satisfaction.

## Author Contributions

All authors made equally significant contributions to the concept, design, and execution of this consensus method manuscript.

## Ethics Statement

The authors have nothing to report.

## Conflicts of Interest

Drs. Akers, Jackson, and McCarthy are employed by Merz Aesthetics, Inc. (Raleigh, NC). Dr. Vachiramon serves as a speaker for Merz Aesthetics, Beiersdorf, and L’Oreal, and as an advisory board member for Beiersdorf, and L’Oreal. Dr. Pavicic is a consultant, investigator, and lecturer for Merz Aesthetics Inc. Dr. Casabona is a consultant for Merz Aesthetics. Dr. Green is a clinical researcher, advisory board member, and speaker for Allergan Aesthetics. Dr. Levine is a consultant and speaker for Allergan and BTL, a consultant and advisor for Galderma and Merz, and a speaker for RVL. Dr. Park serves as a consultant of Merz Aesthetics. Dr. Spada has received personal compensation for consulting, serving on a scientific advisory board, speaking, professional travel/accommodation stipends, or other activities with Merz Pharma. Dr. Muniz is a medical consultant and speaker for Merz Aesthetics. No compensation was given for authorship of this manuscript.

## Data Availability

The authors have nothing to report.
